# Implication of substance use in suicidal or violent behaviours in a first episode psychosis spectrum disorder population : A 45 patients retrospective study

**DOI:** 10.1192/j.eurpsy.2021.1370

**Published:** 2021-08-13

**Authors:** R. Bordas, C. Jourdan, C. Basso, E. Laffont, M. Pujol, L. Lamary

**Affiliations:** Psychiatry, Hospital Ariege Couserans, Saint Girons, France

**Keywords:** schizophrénia, Substance use, violence, Suicide

## Abstract

**Introduction:**

In First Episode Psychosis (FEP), Suicidal Behaviours (SB), Violent Behaviours (VB) and substance use are frequent respectively 10% to 30%, 34.5% and 50% (Pompili et al., 2011), (Tournier et al., 2013). The role of substance use in facilitating SB and VB is described (Large et al., 2011).

**Objectives:**

We aim to evaluate the impact of substance use in FEP patients. Our hypothesis is that substance use is associated with more SB or VB before first admission.

**Methods:**

First admission files of 45 patients diagnosed ICD10 F20 to F29 during the 2013-2018 period were retrospectively studied. SB, VB and substance use (Cannabis, alcohol and opiate/cocaine) before admission were collected. Correlation between SB and VB were tested with cannabis, alcohol, opiate/cocaine use with chi2 Pearson independance test.

**Results:**

The frequencies of suicidal behaviours and violent behaviours were 25 % and 22.7 %. The frequencies of cannabis use, alcohol use, opiate/cocaine use were 56.1 %, 10 % and 16.3 %. A strong significant correlation was found between opiate/cocaine use and violent behaviour, p = 0.011 Chi2 was 6.471 DF 1. No other significant correlations were found.

**Conclusions:**

Suicidal behaviours and violent behaviours are known to be more frequent in psychotic patients with addictive comorbidity. Our french rural hospital retrospective study confirms that violent behaviours in first admission psychotic patients are strongly associated with opiate/cocaine substance use comorbidity.

